# Developmental Bisphenol A Exposure Modulates Immune-Related Diseases

**DOI:** 10.3390/toxics4040023

**Published:** 2016-09-26

**Authors:** Joella Xu, Guannan Huang, Tai L. Guo

**Affiliations:** 1 Department of Veterinary Biosciences and Diagnostic Imaging, Interdisciplinary Toxicology Program, University of Georgia, Athens, GA 30602-7382, USA; joella@uga.edu; 2 Department of Environmental Health Sciences, University of Georgia, Athens, GA 30602-7382, USA; guhuang@uga.edu

**Keywords:** bisphenol A, immunotoxicity, developmental, epigenetics, microbiome, multiple sclerosis, diabetes, allergy, asthma, mammary cancer, bisphenol S

## Abstract

Bisphenol A (BPA), used in polycarbonate plastics and epoxy resins, has a widespread exposure to humans. BPA is of concern for developmental exposure resulting in immunomodulation and disease development due to its ability to cross the placental barrier and presence in breast milk. BPA can use various mechanisms to modulate the immune system and affect diseases, including agonistic and antagonistic effects on many receptors (e.g., estrogen receptors), epigenetic modifications, acting on cell signaling pathways and, likely, the gut microbiome. Immune cell populations and function from the innate and adaptive immune system are altered by developmental BPA exposure, including decreased T regulatory (Treg) cells and upregulated pro- and anti-inflammatory cytokines and chemokines. Developmental BPA exposure can also contribute to the development of type 2 diabetes mellitus, allergy, asthma and mammary cancer disease by altering immune function. Multiple sclerosis and type 1 diabetes mellitus may also be exacerbated by BPA, although more research is needed. Additionally, BPA analogs, such as bisphenol S (BPS), have been increasing in use, and currently, little is known about their immune effects. Therefore, more studies should be conducted to determine if developmental exposure BPA and its analogs modulate immune responses and lead to immune-related diseases.

## 1. Introduction

Bisphenol A (BPA) is used in polycarbonate plastics and epoxy resins, such as food containers and can linings, with human exposure levels measurable in more than 90% of human urine samples in a 2003–2004 national survey and in a more recent 2007–2009 Canadian survey [1,2]. With such a high frequency of exposure in humans, there has been an increasing amount of research published on BPA exposure in utero and through lactation for many possible developmental and reproductive effects, which can lead to an altered immune system and disease development.

BPA exposure is considered mainly from food. The estimated exposure levels are from 0.01–13 μg/kg/day in children and about 4.2 μg/kg/day for adults [3]. However, BPA in fasting adults did not decrease over time with concentrations greater than 12 ng/mL still present in urine after fasting for 6 or 9.5 h [4]. This suggests that BPA exposures from nonfood sources and/or bioaccumulation in human tissue are important factors for BPA exposure. Additionally, BPA has been detected in fetal cord blood (from 0.14–9.2 ng/g), fetal liver (from 1.3–50.5 ng/g), amniotic fluids (from 0.36–5.62 ng/g), placental tissue (up to 273.9 ng/g) and breast milk (up to 1.16 μg/kg) [3,5].

Due to BPA’s ability to bind to estrogen receptors, BPA is classified as an endocrine-disrupting chemical (EDC). EDCs can decrease the survival of immune cells as seen from animal studies and may suppress type 1 helper (Th1) cell immunity or type 2 helper (Th2) cell immunity, leading to a shift in immune balance; an increase in the Th1 response results in a pro-inflammatory response, while a Th2 shift will result in an anti-inflammatory response [6]. The shift in T helper cell populations (Th1, Th2, Th17, etc.) can alter immune responses to antigens and disease profile, contributing to autoimmune diseases (e.g., multiple sclerosis) and allergic diseases (e.g., asthma) [7].

Studies on BPA’s effect on the immune system are mainly from adult exposure and in vitro studies of differentiated cell lines. However, BPA can affect nuclear receptors and non-nuclear receptors in murine placenta with sex-dependent differences, and it passes through the placenta to the fetus likely via passive diffusion [8,9]. Additionally, developmental BPA exposure has been shown to alter the immune responses and risks for some immune-related diseases in offspring.

## 2. Mechanisms of Immune Modulation and Disease Exacerbation Following Developmental BPA Exposure

### 2.1. Receptor Modification

BPA’s effects on the immune system are likely due to its agonistic and antagonistic effects on receptors. BPA can modulate the immune response through binding to various receptors, including nuclear and non-nuclear receptors. Some of the main receptors bound by BPA are estrogen receptors (ERs), estrogen-related receptors (ERRs), aryl hydrocarbon receptor (AhR), peroxisome proliferator-activated receptors (PPARs) and toll-like receptors (TLRs) [10,11]. Of these receptors, ERs, ERRs and TLRs are expressed in the majority of immune cells; dendritic cells (DCs), macrophages, B cells and the T cell subsets Th17 and Treg express AhR; and PPARs are expressed in macrophages, DCs, T cells and B cells [10,12,13]. This allows BPA to act on the receptors of many different immune cells of the innate and adaptive immune systems. However, these results are currently based on adult exposure, and only a few studies have examined ER or ERR modification following prenatal or perinatal exposure.

Activation of ERs can shift the Th1/Th2 balance to a Th2 profile with increased anti-inflammatory cytokines and inhibited antigen-presenting cell (APC) activation, T cell proliferation and pro-inflammatory cytokine secretion when there is a high level of endogenous estrogen. Low or moderate levels of estrogen lead to a shift towards a Th1 profile with ER binding resulting in increased pro-inflammatory cytokines, APC activation and T cell proliferation [14]. The amount of endogenous estrogen, the amount of BPA that it is exposed to and levels of other hormones, including progesterone, will all determine whether the shift from EDC exposure increases the Th1 or Th2 response. Due to the importance of hormone levels, sex and age also play a key role in the outcome from BPA exposure on the immune system.

The sex-specific effects were seen in a study by Miao et al. [15] in which F344 rats exposed to BPA perinatally had increased ERα expression in the F0 and F1 females, while the F0 and F1 males had decreased ERα expression. Miao et al. [15] also found perinatal BPA exposure to F344 rats resulted in a dose-dependent decrease in IL-2, IL-12, IFN-γ and TNF-α in the F0 and F1 generations. This decrease is likely due to BPA’s ability to alter ERα expression, which has been indicated to modulate expression of IL-2 and IFN-γ and other nuclear and hormone receptors [15,16]. These cytokines are important for the inflammatory response and T cell response, particularly the Th1 response.

While BPA’s affinity for ERRs is greater than ERs, less research has been done on BPA’s immunomodulation through ERR interaction [17,18]. ERRs can bind estrogen response elements (EREs) and the ERR-response element (ERRE) resulting in crosstalk between ERs and ERRs [17]. Additionally, ERRs regulate oxidative metabolism and mitochondrial biogenesis in many cells including T cells and macrophages [19,20]. Of particular importance to the adaptive immune system, ERRα regulates T effector cell function and is needed for T effector cell activation and differentiation [21]. In Jurkat cells, a leukemic T cell line, it was found that BPA increased ERRα expression in a concentration-related manner, which suggested that BPA could alter T cell function through modulating ERR expression [20]. Developmental BPA exposure can also alter ERR levels, as seen from low dose (2 and 20 μg/kg) prenatal BPA exposure altering prefrontal cortex and hypothalamus ERRγ gene expression in a sex-specific manner (increasing in males, while decreasing in females) [22]. ERRs are also involved in diseases, for example increased expression contributing to breast cancer proliferation and alleviating EAE, which could be a mechanism for BPA to alter immune-related diseases, as discussed later [19,21].

### 2.2. Epigenetics

Most EDCs including BPA do not directly affect DNA by altering the DNA sequence or causing mutations. Instead, studies show that epigenetic changes are taking place with many EDCs including BPA [6,23,24]. These types of changes have the potential to affect immune cells directly by altering the transcription and translation of immune function-related genes or indirectly by increasing the risk for diseases, such as cancer [24,25,26].

DNA methylation, histone modification and RNA interfering are the three most studied types of epigenetic modifications [27]. DNA methylation results in a more compact and inactive chromatin with less transcription. Histone modifications can result in a more tightly bound nucleosome and less transcription or a more loosely bound nucleosome and more transcription, depending on the modification. The two main types of histone modifications are acetylation, which upregulates transcription, and methylation, which may upregulate or downregulate transcription based on the site of the modification. RNA interfering involves silencing from post-transcriptional RNA, with miRNA being the most studied of these RNAs [27].

Developmental BPA exposure can modify the epigenome through all three mechanisms [23,24]. Although studies focusing on epigenetic effects on the immune system are lacking, other studies on BPA’s ability to alter the epigenome show that immune gene transcription and translation are likely also changed. For example, prenatal BPA can alter DNA methylation patterns in different mouse fetal tissues including fetal liver tissue [28]. Additionally, Dhimolea et al. [23] found DNA methylation and histone modification patterns of the mammary gland epigenome and, specifically, genes involved in cancer (e.g., p. 57), in rats following prenatal BPA exposure change over time differently from control rats. A cohort study of prepubescent Egyptian girls found that higher BPA levels in urine correlate with more hypomethylated genes involved with immunity and inflammation, indicating that BPA increases immune and inflammatory response through epigenetics [29]. Nonetheless, whether epigenetic alterations affect immune genes after developmental exposure in humans has yet to be researched. While further studies are needed to determine BPA’s effect on the immune system from epigenetic alteration, this reported evidence suggests epigenetics as a mechanism for developmental BPA exposure altering the immune system.

### 2.3. Microbiome

One of BPA’s main routes of exposure is oral, resulting in exposure of the gut-associated lymphoid tissue (GALT). The GALT contains both innate and adaptive immune cells that interact with intestinal epithelial cells and microbiota [30,31]. This raises the possibility that BPA can affect the immune system through interaction with the gut microbiota.

Dysbiosis, a microbial imbalance or maladaptation, has been associated with an increase in disease risk including type 1 diabetes (T1D), but the mechanisms are still unclear [32]. Environmental exposures to different chemicals can cause dysbiosis in infants and increase their risk for diseases [31]. To date, there are no studies on BPA’s effect on the gut microbiota. Furthermore, the mechanisms of how environmental exposures to chemicals such as BPA may affect the microbiota and possibly cause dysbiosis that leads to immune dysfunction and disease are still unclear. It is known that the early developmental stages are critical for metabolic maturation, and alterations of the gut microbiota during this period can lead to obesity with an increased risk for diseases like T1D, heart disease and cancer [33]. Dysbiosis also likely results in increased permeability of the intestinal epithelial layer, leading to a leaky gut, which often precedes inflammatory diseases, such as T1D [34], but more studies are needed to determine the interaction between the microbiome and the intestinal epithelial layer [34]. Braniste et al. [35] found that BPA exposure alters the intestinal barrier permeability and function leading to inflammation, which possibly resulted from alterations in the gut microbiota. Due to the close relationship between the GALT and the gut microbiota, these studies suggest that BPA exposure disrupts gastrointestinal function and affects gut microbiota composition, and further research would provide better insight.

### 2.4. Cell Signaling Pathways

BPA can act through cell signaling pathways to alter the immune system and increase disease risk [36]. Early growth response gene-2 (EGR2) and signal transducer and activator of transcription 3 (STAT3) are two examples of cellular processes that BPA can activate to alter immune functions such as inflammation [37,38]. EGR2 is involved in development of Treg cells, dendritic cells and Th17 cells and suppresses naive CD4^+^ T cells [39]. In Treg cells, ERG2 activation increases IL-10 production, while in Th17 cells, ERG2 activation decreases IL-17 production. Additionally, dendritic cell activation of ERG2 decreases IL-12, IFN-γ and antigen uptake [39]. EGR2 also regulates LAG3^+^ Treg cells and is required for T cell anergy [40]. ERG2 modulation of immune cells suggests that BPA activation of this cellular pathway can alter immune responses, leading to ERG2-related diseases including lupus-like syndrome, seen in C57BL/6 mice, and allergic airway diseases, seen in BALB/c mice [40,41].

STAT3 is required for IL-27-mediated EGR-2 induction [40]. Activation of STAT3 downstream effects depends on the microenvironment. STAT3 regulates tumor-associated inflammation, and its activation downregulates the Th1 response by upregulating Foxp3^+^ Treg cells [42,43]. STAT3 regulation of inflammation during *M. tuberculosis* infection differs from tumor-associated inflammation by downregulating Foxp3^+^ Treg cells and macrophage inflammatory response and modulating Th cell response [44,45]. STAT3 can be activated by immune cytokines (e.g., IL-10) and EDCs including BPA [38,42,46]. While studies have shown the activation of STAT3 and EGR2 by BPA, the molecular mechanisms of how these cellular pathways are activated by BPA are unclear. However, the immunomodulation by BPA affecting these pathways can result in increased disease risks, such as cancer [47]. Additionally, other signaling pathways, such as NF-κB and ERK 1/2 phosphorylation in macrophages, can be altered by BPA to dysregulate immune cell function and cytokine production [48,49,50].

## 3. Immune System Alteration Following Developmental BPA Exposure

### 3.1. Innate Immune System

The alteration of the innate immune response from BPA exposure can be seen from zebrafish embryos, which lack an adaptive immune system at early ages. Zebrafish embryos exposed to varying concentrations of BPA (0.44–4400 nM) developed an innate immune response resulting from oxidative stress as seen from increased reactive oxygen species (ROS) and downstream effects of upregulated TLRs, cytokines and chemokines, inducible nitric oxide synthase (*iNOS*) and nitric oxide (NO) [51]. This study shows that BPA can affect the innate immune system and signaling for upregulated pro-inflammatory (e.g., INF-γ) and anti-inflammatory markers (e.g., IL-10). In addition, this study suggests that BPA alters macrophages and neutrophils as seen from the upregulated expression of cytokines and chemokines produced from these cells.

Studies using macrophage cell lines and a perinatally-exposed diabetic mouse model have shown that the number of macrophages is decreased from BPA exposure with higher apoptotic cells [52,53,54]. Macrophage functions are also altered by BPA as seen from both cell models and developmental mouse studies. BPA can decrease phagocytosis activity of macrophages, which possibly reduces the immune defense against pathogens [49,52,54]. Additionally, BPA can increase inflammation by increasing macrophage production of pro-inflammatory cytokines (e.g., TNF-α) and decreasing anti-inflammatory cytokines (e.g., IL-10) [48,49,52]. However, BPA has also been found to decrease macrophage pro-inflammatory cytokine production at very high concentrations, which are less physiologically relevant, suggesting that the amount of BPA that it is exposed to determines its effects on macrophages [50,54,55].

Eosinophils, which are involved in allergy and asthma development, can also be altered from developmental BPA exposure. BPA in combination with allergen exposure (e.g., OVA) differently modifies eosinophil production by increasing eosinophils from early life exposure and decreasing eosinophils from late life exposure as discussed in the section below on allergies and asthma [56,57,58]. These studies suggest that not only the doses of BPA that it is exposed to, but also that the windows of exposure play a role in the modulation of the innate immune system.

### 3.2. Adaptive Immune System

BPA exposure has been found to decrease Treg cells, which are important for immune cell balance, resulting in an altered Th1/Th2 response [10]. Prenatal BPA exposure in both male and female mouse offspring can increase CD3^+^CD4^+^ (T helper) and CD3^+^CD8^+^ (cytotoxic T cells) splenic T cell populations [59]. Increases of these T cell populations could be a result from decreased Treg cells, which are important for regulating T helper and cytotoxic T cell functions. The unregulated increase in these T cell populations (e.g., autoreactive CD8^+^ and CD4^+^ T cells) increases the risk for immune-related diseases [60,61,62].

Chick embryos exposed to BPA exhibited altered structures of the thymus, where T cells develop, and bursa of Fabricius, where B cells develop in birds [63]. However, no studies yet have examined the effects of prenatal or perinatal BPA exposure on bone marrow, where B cells develop in mammals. Structural alteration of organs where T and B cells develop can negatively affect the adaptive immune cell development and may result in altered cell populations and/or functions [64,65], which may further increase susceptibility to infections and diseases [60,66].

### 3.3. Cytokine/Chemokine, Antibody Production and Host Resistance

Alteration of cytokines and chemokines by BPA might result from alterations in the number of immune cells, expression of genes or receptor modifications in immune cells. Due to BPA’s effect on ER, cytokines and chemokines are likely affected in both the innate and adaptive immune system. Holladay et al. [67] observed that C57BL/6 male offspring perinatally exposed to 1.0 mg/kg BPA had a trend of upregulated cytokines and chemokines towards a Th1 and Th17 response at 20 weeks old; however, this study lacked enough mice for statistical analysis, and the dose was high. Similar results were found from measuring the immune response in 300 and 3000 μg/kg prenatally BPA exposed male offspring in mice that were immunized with hen egg-white lysozyme (HEL) protein at eight and 11 weeks old when both INF-γ and IL-4, Th1 and Th2 cytokines, respectively, were increased along with increased anti-HEL IgG titers; however, the Th1 response had a higher increase [59].

In another study that measured markers for allergy and inflammatory processes in human cord blood and compared the amount of maternal urinary BPA concentration during the first trimester, it was found that thymic stromal lymphopoietin (TSLP), IL-33 and IgE levels were elevated in a non-linear manner in both males and females exposed to BPA during fetal development, suggesting that both low and high exposure levels of BPA during the first trimester might result in allergies or inflammatory diseases [68]. The non-linear response seen in this study is common for many EDCs, including BPA, and has resulted in increased concerns over human exposure, which tends to be lower than the doses used in experiments [69,70]. Together, these studies suggest that developmental BPA exposure can alter the production of mediators important for immune function, which can shift the immune response in a non-monotonic manner.

Male mice prenatally exposed to BPA had decreased Treg cells along with increased Th1 and Th2 cytokine markers INF-γ and IL-4, respectively, after being challenged with *L. major* protozoa infection [71]. In contrast, perinatally-exposed female Wistar rats exhibited no change in INF-γ, IL-4 or anti-OVA antibody titers following ovalbumin (OVA) antigen challenge; however, Treg cells, T helper cells and dendritic cells were significantly decreased in the spleen and mesenteric lymph nodes (MLN) [72]. In the same study, female offspring following challenge with nematode intestinal infection showed no change in IgE, but increased survival of larvae from decreased neutrophils and myeloperoxidase (an enzyme produced by neutrophils), along with increased Th2 cytokines IL-13 and IL-4, as well as pro-inflammatory cytokines growth-related oncogene and INF-γ, in the small intestine [72]. The differences between these results suggested the antigen-specific effects of BPA in altering the immune system, which could be a downstream effect from decreased Treg cells by disrupting the immune cell balance after BPA exposure.

In contrast, perinatally BPA exposed male and female mice showed no difference in virus-specific IgG levels, cytotoxic T cells, T helper cells or Treg cells following challenge with influenza A virus infection; however, a decrease in cytokines and chemokines produced by neutrophils and macrophages similar to the other host resistance studies as discussed above was observed during early infection [73]. This suggests that the adaptive immune response to viral infection is not affected by BPA exposure, but the innate immune response may be delayed as seen from downregulated cytokines and chemokines produced by neutrophils and macrophages during early infection. The differences in BPA’s effect on T cells between these studies could be from different antigens used, species/strains used, sexes of animals used and from different doses. Additionally, the different host resistance tests that measured innate immune response tended to show a decrease in innate immune cells along with downregulation of cytokines and chemokines produced by innate immune cells.

## 4. Diseases Related to Immune System Alteration Following Developmental BPA Exposure

### 4.1. Multiple Sclerosis

Multiple sclerosis (MS) is a chronic autoimmune disease resulting from demyelination of nerve cells. T cells, antibodies and inflammatory responses all contribute to MS development. CD8^+^ T cells and macrophages are the predominant immune cells involved in human MS lesions. BPA is likely to have neurotoxic effects, which can result in exacerbation of neurological diseases and disorders. Neurotoxic mechanisms of chemicals are suggested to include oxidative stress, epigenetic effects, endocrine disruption and, possibly, lipophilic exogenous chemicals crossing the blood brain barrier (BBB) and allowing the entry of hydrophilic chemicals; BPA has been shown to cause endocrine disruption and epigenetic modification and is lipophilic, enabling BPA to cross the BBB [53,74]. Moreover, ERβ knockout mice have deficits in synaptic plasticity and in neurogenesis, and perinatal BPA exposure inhibits ERβ expression and can activate the *N*-methyl-d-aspartate (NMDA) receptor in hippocampal cells from mice [75]. Whether BPA affects MS warrants further study, since there is a paucity of research published on BPA’s effect on this disease (Table 1). Animal models include autoimmune encephalomyelitis (EAE) for mammals, virus-induced demyelination (Theiler’s murine encephalomyelitis virus (TMEV)) for mice and toxin-induced demyelination for mammals [76].

Krementsov et al. [77] have reported that perinatal exposure to BPA at a dose that produces similar amounts of accumulation as humans resulted in no significant changes in EAE severity from the control in male and female mice on a phytoestrogen-free diet. Moreover, perinatally-exposed adult female C57BL/6J mice, but not SJL/JCrHsd mice, on a phytoestrogen-free diet had decreased IL-17 production [77], which suggested that BPA might be protective, because a reduction in IL-17 ameliorated this disease in mice and, possibly, in humans [78]. IL-17 is a pro-inflammatory cytokine that promotes inflammation in MS lesions and autoimmunity, and downregulation of IL-17 can ameliorate MS [78,79]. Modulation of IL-17 could mean ERG2 is an important part of MS development from BPA exposure, since downregulation of ERG2 has been seen with more severe EAE in mice and in MS patients [39].

On the other hand, Brinkmeyer-Langford et al. [80] using the TMEV model of this disease found a significantly increased onset of disease after perinatally exposing male and female mice to BPA. These perinatally BPA exposed mice also had less antibody production to the virus along with increased inflammation of the spinal cord, increased inflammatory colitis, downregulation of anti-inflammatory genes and upregulated IFN-γ, which all lead to a pro-inflammatory response and disease onset [80]. The opposite results in these two studies may be due to the different multiple sclerosis mouse models and/or phytoestrogen levels in the diet. While both MS models have their pros and cons, EAE is mainly CD4^+^ T cell mediated, while TMEV is mainly macrophage mediated and more similar to MS disease development in humans [76]. This would suggest that BPA’s differential effects based on models may be from BPA altering macrophage function to induce disease in the TMEV model, but not affecting the CD4^+^ T cells in the EAE model. Further research directly measuring these immune cells in these animal models would provide a clearer answer on whether BPA can increase MS risk.

### 4.2. Type 1 Diabetes Mellitus

T1D is an autoimmune disease involving the destruction of pancreatic β-cells. Worldwide, there is an annual increase in T1D incidence by 3%–5% with over a million people affected by this disease in the United States alone [56]. There has been a parallel increase in EDC exposure and incidence of T1D. Exposure to BPA has been associated with an increased incidence of T1D in adult animals and diabetes in humans [81,82]. Whether BPA can increase the risk for T1D from perinatal or prenatal exposure is still uncertain. So far, there has only been two studies published using a T1D mouse model of females exposed perinatally only or throughout life (Table 2), which found that BPA increased the percent diabetes at 20 and 25 weeks of age, respectively, while also increasing apoptosis of macrophages, β-cells and α-cells in pancreatic islets and increasing Foxp3^+^ Treg cells [52,83]. Increased apoptosis of β-cells, α-cells and macrophages is a common factor involved with T1D disease development [57,58]. As discussed earlier, macrophage function was also altered with decreased phagocytic activity, which could result in increased inflammation due to less clearance of apoptotic cells [52]. Additionally, BPA altered splenocyte cytokine production including decreased IL-10, which was likely a mechanism of increased T1D development from BPA exposure [52]. In the perinatal exposure only study, however, these effects were only seen at a very high dose (10 mg/L; about 3000 μg/kg/day), while lower doses (about 30 and 300 μg/kg/day) did not show much effect, although the effects were seen at a lower dose (1 mg/L) in the lifelong study.

### 4.3. Type 2 Diabetes Mellitus

Increasing evidence suggest that type 2 diabetes (T2D) is also related to immune dysfunction [84]. Increasing exposure to EDCs is suspected as a contributive factor to T2D along with known causes, such as obesity, decreased insulin sensitivity and disrupted β-cell mass and function [85,86]. Chronic inflammation produced by obesity plays a key role in T2D development with altered immune cell populations (e.g., CD4^+^ Tregs) and insulin resistance contributing to the disease development [84].

A birth cohort study observed a correlation between higher BPA levels in Canadian mother’s urine during the first trimester and lower adiponectin levels in cord blood at term birth of male metabolic syndrome and T2D in adulthood [85]. While this cohort had a large participation (2001 women), the ethnic groups were limited with mainly Caucasian women participating. Another potential bias could result from only one time point for measuring BPA exposure, since infants were likely exposed to varying amounts throughout infants, but not female infants; low adiponectin levels at birth are a risk for insulin resistance gestation. 

In agreement with the cohort study, animal studies also show a higher risk for male offspring, but not as much for female offspring, as seen by increased body weight, increased insulin levels and insulin resistance from BPA exposure [87,88]. Although glucose levels and glucose tolerance only tend to be affected in males on phytoestrogen-free diets and in both sexes on high-fat diets following BPA exposure, T2D is characterized more by increased insulin levels and insulin resistance [86,87,88,89,90]. BPA exposure alters β-cell mass and function by reducing β-cell turnover and proliferation and increasing β-cells containing swollen mitochondria and rough endoplasmic reticulum in male offspring and in both sexes on a high-fat diet [86,88].

**Figure 1:**
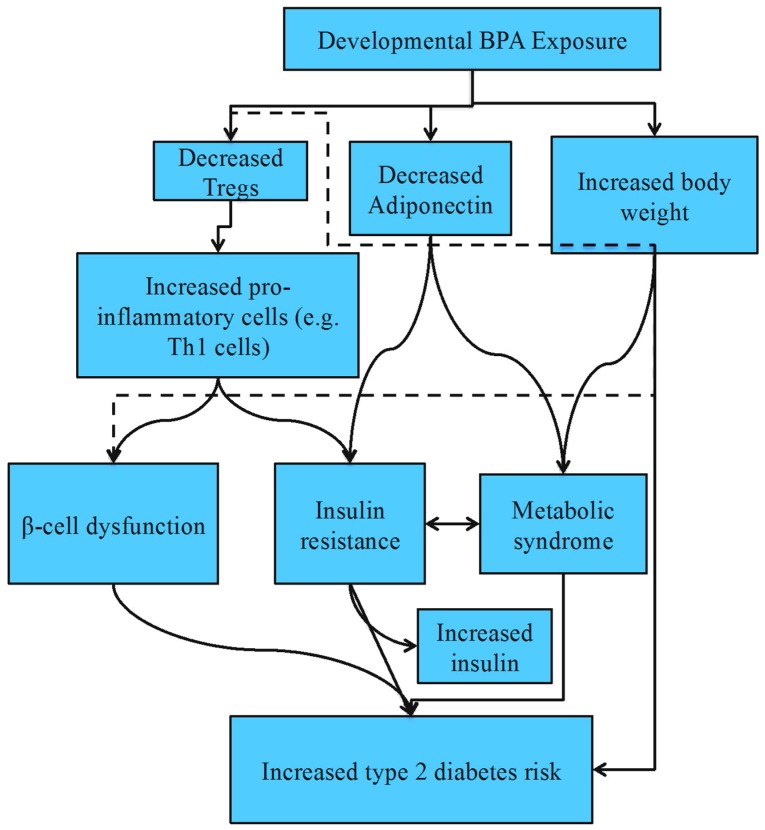
Proposed mechanisms for increased type 2 diabetes risk following developmental bisphenol A (BPA) exposure.

Both animal studies and the previously-mentioned infant cohort study identify prenatal and perinatal BPA exposure as a risk factor for T2D development in males on phytoestrogen-free diets and in both sexes on a high-fat diet (Table 2; Figure 1). The immune system plays an important role in T2D. Obesity increases the accumulation of pro-inflammatory immune cells (e.g., macrophages, cytotoxic T cells and Th1 cells), while decreasing Foxp3^+^CD4^+^ Treg cells [84]. Inflammation resulting from the increased pro-inflammatory cell environment disrupts insulin action and produces insulin resistance and β-cell dysfunction [84]. As discussed, developmental BPA exposure can increase the Th1 response and reduce Treg cells, which is likely a mechanism for the increased T2D seen in these studies.

Additionally, some epidemiological studies found higher BPA and chlorinated BPA levels during adulthood correlated with increased type 2 diabetes risk [82,91,92]. However, some other epidemiological studies in adults found no correlation, which was likely due to differences in genetic susceptibility modulating BPA’s effects and other confounding factors [92,93,94,95]. These adult epidemiological studies show the importance of measuring genetic susceptibility in future studies to examine the relationship between developmental BPA exposure and T2D development, which has also been highlighted in an animals study [96].

### 4.4. Allergies and Asthma

About one generation after widespread use of BPA, childhood asthma prevalence started increasing [97]. Allergies and asthma generally result from an imbalance in the Th1/Th2 ratio with a shift towards a Th2 response [98]. Additionally, newborns tend to have a Th2-biased immune system, so chemicals such as BPA that increase the Th2 response likely increase the risk for allergies and asthma in childhood [97]. Immune cells involved in the pathogenesis of allergies and asthma include T cells, mast cells, eosinophils, macrophages and B cells [98,99]. Although epidemiological studies have not made a clear conclusion concerning BPA increasing the risk of allergy and asthma, BPA exposure likely increases risk by altering immune cell function and cytokine production towards a Th2 response [100].

Animal studies using ovalbumin (OVA) to elicit an allergic immune response after either perinatal or prenatal BPA exposure have found different results depending on the method of OVA sensitization, sex and time of exposure. Overall, for female developmental exposure in rodents, BPA shows a trend for increased risks of allergies and asthma after sensitization with antigen, while male rodent offspring have a mixed response depending on the route and timing of antigen exposure (Table 3). Human epidemiological studies tend to show increased asthma symptoms in children developmentally exposed to BPA, but no effect on allergic responses induced by other common allergens (e.g., cat epithelium; Table 4).

**Figure 2:**
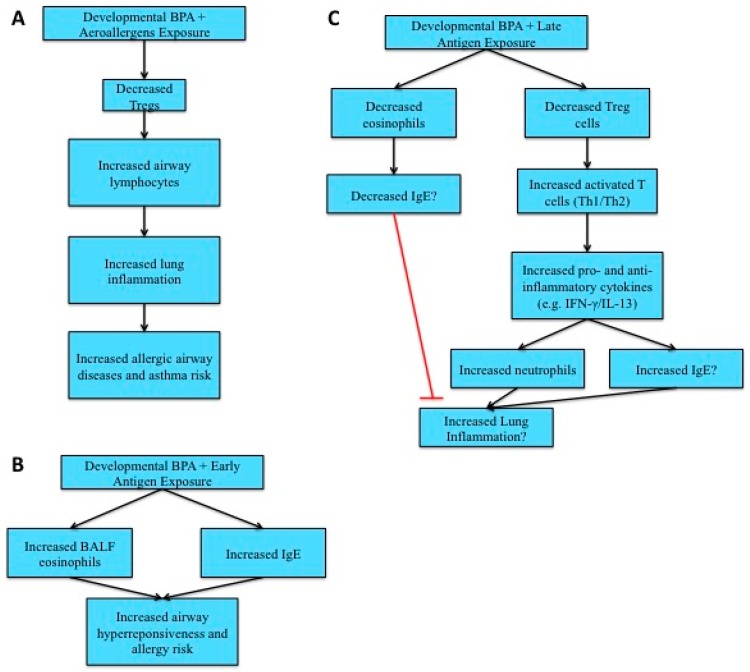
Allergy and asthma risks following developmental bisphenol A (BPA) exposure in female rodents. (**A**) Increased allergic airway and asthma risks result from developmental BPA and aeroallergen exposures; (**B**) increased airway hyperresponsiveness and allergy risks result from developmental BPA and early life antigen exposures; (**C**) lung inflammation may result from developmental BPA and late antigen exposures, but this is uncertain.

Female offspring: Airway OVA sensitization of perinatally BPA exposed female offspring have increased lymphocytes in airways along with increased inflammation when compared to OVA-sensitized control female offspring, but no changes in T cell subpopulations or anti-OVA IgE [101]. This study suggests that perinatal BPA exposure can increase the risk for asthma and allergic airway diseases by increasing lymphocytes in airways, leading to increased lung inflammation after exposure to aeroallergens (Figure 2A). Similarly, following a “suboptimal” peritoneal sensitization (e.g., exposing female pups to a lower OVA dose at an earlier age), perinatal BPA exposure results in increased airway hyperresponsiveness (AHR), bronchoalveolar lavage fluid (BALF) eosinophil and anti-OVA IgE and no change in anti-OVA IgG1 compared to OVA-sensitized control females [97,102]. This study confirms that developmental BPA exposure can increase female offspring’s risk for allergies and asthma after allergen sensitization (Figure 2B).

In the above studies, increased IgE and BALF eosinophils result in AHR. In contrast, research using a standard peritoneal sensitization procedure shows that female offspring exposed perinatally to BPA have decreased eosinophils [101,103]. Additionally, BPA’s modulation of anti-OVA IgE is not consistent, with increased amounts, decreased amounts and no change seen by various studies [101,103,104,105]. However, a trend for increased allergy and asthma risks for perinatal BPA exposed female offspring is still seen (Figure 2C). Perinatal BPA exposure increases IFN-γ, activated T cells, neutrophils and IL-13 in female offspring, which can potentially enhance an allergic response [103,104,105]. However, whether these changes increase lung inflammation is unclear, with some studies suggesting increased lung inflammation while others showing no change in both lung inflammation and AHR [101,103,104]. In summary, all three models of sensitization suggest that developmental BPA exposure increases allergy and asthma risk in female offspring, with early exposure to allergens being the most sensitive exposure window.

**Figure 3:**
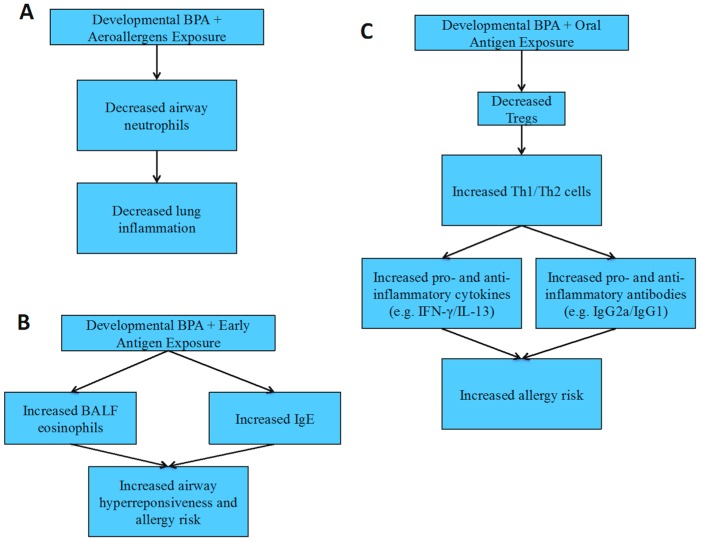
Allergy and asthma risks following developmental bisphenol A (BPA) exposure in male rodents. (**A**) Decreased lung inflammation results from developmental BPA and aeroallergen exposures; (**B**) increased airway hyperresponsiveness and allergy risks result from developmental BPA and early life antigen exposures; (**C**) increased allergy risks result from developmental BPA and oral antigen exposures.

Male offspring: Unlike female offspring, perinatal BPA exposure has a protective effect in airway-sensitized male offspring (Figure 3A). This is reflected by decreased airway neutrophils and lung inflammation, with no change in T cell subpopulations, BALF cytokines or anti-OVA IgE in male offspring exposed to BPA [101]. This study has indicated that BPA’s downregulation of neutrophils causes the decrease in lung inflammation in this model.

Conversely, “suboptimal” sensitization results in an increased risk for allergies and asthma after both prenatal and perinatal BPA exposure in male offspring similar to female offspring (Figure 3B). Both increased BALF eosinophils and anti-OVA IgE increase AHR [97,102]. It seems that the “suboptimal” model is more sensitive for BPA exposed male offspring, as similar results are also seen in the female offspring. While the standard peritoneal sensitization shows no effect in prenatally-exposed BPA male offspring, perinatally BPA exposed male offspring exhibit mixed results with some studies seeing an increase in lung inflammation and inflammatory factors (e.g., IL-13), while others show decreased lung inflammation and inflammatory factors (e.g., eosinophils) [103,104].

Additionally, BPA exposed male offspring show a decreased Treg response to OVA while increased Th1 and Th2 responses, including increased IL-13, INF-γ, anti-OVA IgG1 and anti-OVA IgG2a following OVA sensitization by gavage, suggesting increased allergy and asthma risk (Figure 3C) [99]. These studies show that the route and timing of antigen/allergen exposure would determine whether BPA exposed male offspring are at an increased or decreased risk for allergies and asthma. The differences seen in the males versus the females are likely due to the differences in hormone levels (e.g., estrogen).

**Figure 4:**
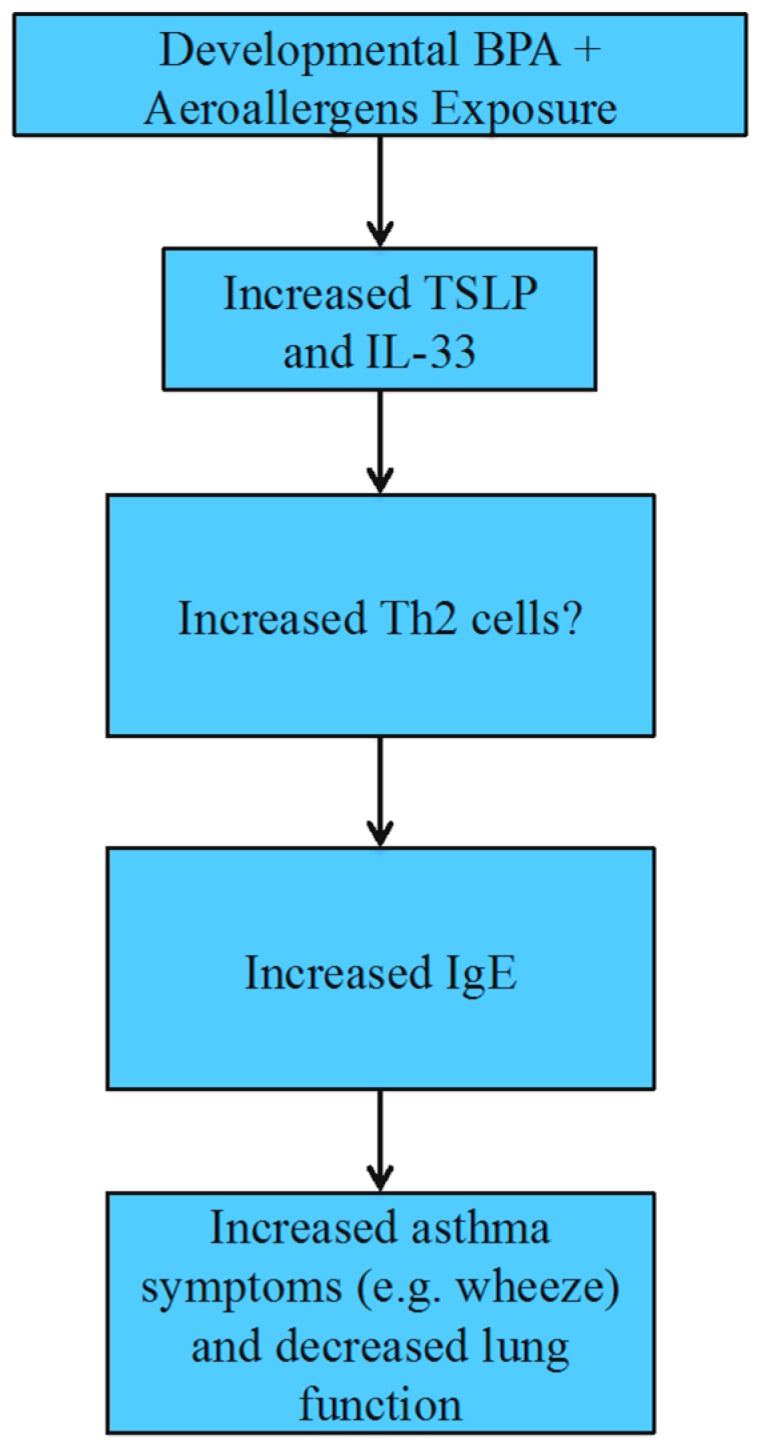
Asthma risk increases following developmental bisphenol A (BPA) and aeroallergen exposures in humans.

Epidemiological studies: Human exposure to BPA during any of the three trimesters results in increased asthma biomarkers and symptoms (Figure 4). However, allergy sensitivities to antigens other than aeroallergens as measured by IgE-specific seroatopy are not affected [107,108]. Higher BPA levels result in increased TSLP, IL-33 and IgE from cord blood, which are biomarkers for allergies and asthma development later in life [68]. Additionally, increased BPA exposure increases asthma outcomes, including wheeze, persistent wheeze, respiratory tract infection and bronchitis, up to the age of seven years old and decreases lung function until four years of age [108,109]. These cohort studies suggest that asthma risk increases in children who have a higher than average prenatal BPA exposure. Although most epidemiological studies show exacerbation of asthma symptoms from higher prenatal BPA exposure, Donohue et al. [107] found that higher prenatal BPA levels result in a protective effect by decreasing wheeze. The difference in asthma outcome seen from prenatal BPA exposure by Donohue et al. [107] could be due to the cohort design differences or difference in sample populations. However, Donohue et al. [107] also found increased risks of asthma, wheeze, airway inflammation and aeroallergen sensitization from higher postnatal BPA levels up to seven years old.

### 4.5. Mammary Cancer

Both the innate and adaptive immune systems play important roles for primary and recurring mammary cancer prevention along with tumor growth and metastasis [42,43,110]. Additionally, in utero exposure to endocrine disruptors has long-term effects on mammary tissue, and the fetal origins of cancer hypothesis has developed from studies focusing in EDCs including BPA [111]. Most research on developmental BPA exposure and cancers focuses on mammary cancer due to BPA’s interaction with ERs [112,113]. However, alteration of the immune system by BPA may increase the risk of other cancers, including an increase in hepatic adenomas, as seen by Weinhouse et al. [112]. Developmental BPA exposure can alter mammary tissue structure and development and increase hormone responses through ERs, leading to increased risk of mammary cancer [114,115]. Additionally, prenatal and perinatal BPA exposure can alter mediators related to immunity and defense in the mammary gland, increasing the susceptibility of mammary gland cancer cell transformation in female offspring [114,116,117]. BPA alters mammary gland ER expression by decreasing ERα, while increasing ERβ. Perinatal BPA exposure also decreases chemokines that further result in decreased attraction of immune cells, such as neutrophils and lymphocytes to mammary gland tissue to remove abnormal cells [114]. Decreased inflammation can decrease immune defense against pre-cancerous and cancerous cells [118]. Furthermore, immune cell and cytokine dysfunction resulting from developmental BPA exposure can increase the risk for mammary cancer [114].

## 5. Bisphenol S: An Alternative for BPA

Bisphenol analogs, such as bisphenol S (BPS), have been increasing in use as BPA use decreases. BPS was found in 81% of analyzed urine samples from the U.S. and seven Asian countries with concentrations up to 21.0 ng/mL from a 2010–2011 study [119]. Although BPS has a widespread use, there is a lack of research on any developmental effects on the immune system from BPS exposure. However, BPS also seems to have a low-dose effect similar to BPA and has been found to adversely affect neurogenesis of zebrafish and Wistar rats [120,121]. BPS can also alter gene transcription in a human osteosarcoma cell line related to immunity (e.g., downregulating CCL2) [122]. However, research on developmental BPS exposure’s effects on the immune system is needed to draw a conclusion.

## 6. Discussion

BPA alteration of the immune response varies by exogenous and endogenous factors, such as diet, estrogen levels and genetic differences [95,96]. This can result in a Th1 or Th2 shift, although a Th1 shift towards a pro-inflammatory response is more likely. BPA reduction of Treg cells is likely one of the main reasons that both Th1 and Th2 cells can increase from BPA exposure, which can lead to altered immune function and disease development. BPA can also increase the risk for diseases by reducing the number of APCs, such as macrophages. Moreover, studies performed after weaning when BPA was no longer present have shown the lasting alteration of immune function.

BPA’s main mechanisms of immunomodulation and disease development are through interactions with both nuclear and non-nuclear receptors, but primarily ERs and ERRs. Additionally, developmental BPA exposure can alter gene transcription through epigenetic mechanisms and likely also alters transcription and translation of genes related to the immune system. More research examining the effect from developmental exposure to BPA on the epigenome with relation to immunity is needed. A third possible mechanism of immunomodulation is via interaction with microbes in the gut microbiome. The importance of the gut microbiota in immunity and disease has been emerging recently. There have yet to be any studies examining if BPA alters the gut microbiota causing dysbiosis. Due to the close proximity of the GALT and gut microbiota, dysbiosis may also be a mechanism of developmental BPA exposure in immunomodulation, though no conclusions can be made yet.

Alteration of the immune system may result in neurological diseases from BPA exposure due to its ability to cross the BBB. There is some research relating BPA’s effect on multiple sclerosis, but the results show either no effect (in the T helper cell-mediated disease model) or an exacerbated effect (in the macrophage-mediated disease model). These studies indicate that primary immune cells mediating this disease in humans affect whether BPA can exacerbate multiple sclerosis. More studies are needed to understand the etiology of MS.

Diabetes is another diverse disease, the immune alterations of which can increase the risk for both type 1 and type 2 diabetes. BPA exacerbated T1D development in prenatally-exposed females. This effect was due to BPA reducing macrophage cells, β-cells and α-cells, which all result in increased T1D risk. However, the increased risk seen in females was only at a high dose not as relevant for human exposure and only observed at a more relevant dose when BPA exposure was throughout life. In addition, whether males are at risk has not yet been researched. As for T2D, an increased risk has been shown for T2D development after prenatal and perinatal exposure to BPA in male offspring and in both sexes on a high-fat diet. While females did not show an increase for T2D risk in the absence of a high-fat diet, feeding a high-fat diet models human T2D development better than from genetic diversity alone [123]. BPA’s increase of T2D risk results from an increase in a pro-inflammatory response and dysregulated β-cell function, which produces insulin resistance, further increasing diabetes risk.

As for asthma and allergy development after developmental BPA exposure, BPA likely increases asthma risk. However, allergy risk seems to only increase in rodent studies and not in epidemiological studies. Furthermore, developmental BPA exposure has a protective effect on lung inflammation in male rodent offspring, which indicates a protection against asthma and allergic airway diseases. This was opposite from male children, which had increased asthma symptoms. The differences are likely due to the differences in the species, such as genetics and hormone concentrations. Developmental BPA also can increase cancer risk for different cancers, including mammary cancer and liver cancer, by altering the immune and inflammatory response. However, it should be noted that, unlike studies using other disease models, the inflammatory response was decreased from developmental BPA exposure in the cancer models used. This may be due to BPA producing its cancer-promoting effects through the STAT3 pathway [47], which upregulates Foxp3^+^ Treg cells and downregulates the Th1 inflammatory response in the tumor microenvironment [42].

In conclusion, BPA alteration of the immune system after prenatal and perinatal exposure has been shown to result in a shift in the response of T helper cells. This can lead to neurological and autoimmune diseases. However, there is still a paucity of research on the final disease outcome from developmental BPA exposure for many diseases, and this warrants further study due to BPA’s known immune effects. In addition, the changes in the immune system and increased risk for different diseases from developmental BPA exposure might also be a concern for BPS. BPA analogs such as BPS have been increasing as BPA decreases in manufacturing. However, little is known about the immunomodulatory effects of BPS, which is a concern considering the increasingly widespread use of BPS, and more research is needed.

## Figures and Tables

**Table 1:** Summary of animal multiple sclerosis studies on BPA exposed offspring.

Multiple Sclerosis Disease Models	Animal Model	Exposure Windows	BPA Dose	Routes of Administration	Diet	Effects	Reference
Autoimmune Encephalomyelitis (EAE)	Male and Female Mice (C57BL/6J and SJL/JCrHsd)	Gestation and Lactation	10 μg/mL	1% ethanol in drinking water	AIN-93G (casein-based phytoestrogen-free)	No Effect on EAE or INF-γ; decreased IL-17 in females	[77]
Theiler’s Murine Encephalomyelitis Virus (TMEV)	Male and Female Mice (SJL)	Gestation and Lactation	10 μg/kg BW	Charcoal-stripped corn oil via gavage	Not Specified	Earlier onset of disease; increased inflammation; decreased antibodies to virus	[80]

BPA: bisphenol A; BW: body weight.

**Table 2:** Summary of animal diabetes (type 1 and type 2) studies on BPA exposed offspring.

Diabetes Disease Model	Animal Model	Exposure Windows	BPA Dose	Routes of Administration	Diet	Effects	Reference
**Type 1 Diabetes**	Female NOD/ShiLtJ Mice Offspring	Gestation and Lactation	0.1, 1 or 10 mg/L	Deionized autoclaved drinking water	2919X (minimal phytoestrogen content)	Increased insulitis, diabetes, Treg cells and apoptosis of β-cells, α-cells and macrophages in highest dose only	[83]
**Type 1 Diabetes**	Female NOD/ShiLtJ Mice Offspring	From Gestation to End of Study	1 mg/L	Deionized autoclaved drinking water	2919X (minimal phytoestrogen content)	Increased insulitis, diabetes and apoptosis pancreatic cells macrophages; decreased phagocytic macrophages, IL-10, IL-4 and TNF-α	[52]
**Type 2 Diabetes**	Male and Female Human Infants	1st Trimester	≤0.34 to >1.7 μg/L (measured, not dosed)	Measured exposure from environment, etc.	Not Specified	Lower adiponectins in male cord blood	[85]
**Type 2 Diabetes**	Male Wistar Rat Offspring	Gestation and Lactation	50 μg/kg	Gavage; dissolved in corn oil	Not Specified	Increased insulin and insulin resistance; reduced glycogen	[87]
**Type 2 Diabetes**	Male and Female Wistar Rat Offspring	Gestation and Lactation	50, 250 or 1250 μg/kg	Gavage; in corn oil	Standard or high-fat diet	Low dose only: increased body weight and insulin; altered β-cell function; high-fat diet and male had a greater effect	[88]
**Type 2 Diabetes**	Male and Female OF-1 Mice Offspring	Prenatal (GD9-16)	10 or 100 μg/kg	S.C. injection; in tocopherol-stripped corn oil	Soy/alfalfa-free	Low dose, males only: increased insulin, insulin sensitivity and glucose intolerance; altered β-cell function	[89]
**Type 2 Diabetes**	Male and Female CD-1 Mice Offspring	Gestation and Lactation	About 0.25 μg/kg	In food	Phytoestrogen-free until weaning then LFD and half mice after 9 weeks old high-fat diet	No effect on glucose tolerance	[90]
**Type 2 Diabetes**	Male OF-1 Mice Offspring	Prenatal (GD9-16)	10 μg/kg	S.C. injection; in tocopherol-stripped corn oil	Soy/alfalfa-free	No effect for insulin sensitivity; glucose intolerance; increased NEFA	[86]

Treg cells: T regulatory cells; GD: gestation day; LFD: low butter-fat diet; S.C.: subcutaneous; NEFA: non-esterified fatty acids.

**Table 3:** Summary of allergy/asthma animal studies on BPA exposed offspring.

OVA Sensitization	Animal Model/Sex	Exposure Windows	BPA Dose	Routes of Administration	Diet	Effects	Reference
Airway Sensitization	Male and Female C57BL/6 Offspring	GD6-PND21	0.5, 5, 50 or 500 μg/kg	Peanut oil via gavage	AIN76-semi-PD1RR chow (phytoestrogen-free)	Increased airway lymphocytes and lung inflammation in females; decreased airway neutrophils and lung inflammation in males; no effect on IgE, T cell subpopulations or BALF cytokines	[101]
“Suboptimal” Peritoneal Sensitization	BALB/c offspring	Gestation and Lactation	5 or 10 μg/mL	Drinking water	Phytoestrogen-free	Increased AHR, BALF eosinophils and IgE; no effect for IgG1	[97]
	BALB/c offspring	Prenatal, perinatal or postnatal	5 μg/mL	Drinking water	Phytoestrogen-free	Increased AHR and BALF eosinophils from prenatal and perinatal; no effect for postnatal only exposure	[102]
Peritoneal Sensitization	Female C57BL/6 Offspring	GD6-PND21	0.5, 5, 50 or 500 μg/kg	Peanut oil via gavage	AIN76-semi-PD1RR chow (phytoestrogen-free)	Decreased airway eosinophils and IgE; no effect on AHR	[101]
	Male and Female BALB/c Offspring	Gestation and Lactation	50 ng, 50 μg or 50 mg/kg diet	In food	AIN-93G (phytoestrogen-free)	Increased IgE, IL-13 and INF-γ; decreased BALF leukocytes, eosinophils, IL-17 and CysLTs; decreased macrophages, PMN and lung inflammation in males; in females only: decreased BALF IL-4, IL-13 and TNF-α, increased lung RANTES and no effect on lung inflammation	[103]
	BALB/cByJ Offspring	One week after mating period until birth or PND21	5 μg/mL	Drinking water	C1000 (phytoestrogen-free)	Prenatal: no effect on AHR or airway inflammation; perinatal: increased lung inflammation, IgE and IL-13	[104]
	Female Wistar rats offspring	GD15-PND21	0.5, 5 or 50 μg/kg	4% ethanol in corn oil via oral	Rodent Diet 2018 (<20 pmol estrogen content)	Increased IgG, activated T cells, splenocyte proliferation, INF-γ, neutrophils and IL-10 (colon); no effect for IgE, Treg cells or IL-10 (spleen); decreased TGF-β (colon)	[105]
Gavage Sensitization	Male heterozygous offspring of OVA-TCR-Tg crossed with BALB/c	Gestation and Lactation	0, 0.1 or 1 ppm BPA	In Food	Not Specified	Increased IL-13, INFγ, anti-OVA IgG1 and anti-OVA IgG2a; no change in IL-4; decreased OVA-specific T cells and Treg response to OVA	[99]

BPA: bisphenol A; OVA: ovalbumin; GD: gestation day; PND: postnatal day; BALF: bronchoalveolar lavage fluid; AHR: airway hyperresponsiveness; CysLT: cysteinyl leukotriene; PMN: polymorphonuclear neutrophil; Treg: T regulatory cells.

**Table 4:** Summary of allergy/asthma epidemiological studies from BPA exposed infants/children.

Sex/Age	Time of BPA Measurement	BPA Measured	BPA Levels Assessed From	Effects	Reference
Male and Female Infants	1st Trimester	0.8 μg/L	Median urine concentration	Non-monotonic increase of TSLP, IL-33 and IgE in cord blood	[68]
Male and Female Children	16 weeks gestation, 26 weeks gestation and birth	2.4 μg BPA/g creatinine	Median urine concentration	Increased wheeze risk of 6 months old, but not 3 years	[106]
Male and Female Children	3rd trimester, 3, 5 and 7 years old	1.8 ng/mL (3rd trimester), 3.8 ng/mL (3 years), 3.1 ng/mL (5 years), 2.7 ng/mL (7 years)	Median urine concentration	Higher prenatal BPA levels inversely correlated with wheeze at 5 years and bronchodilator response; postnatal exposure increased wheeze, airway inflammation and aeroallergen sensitization at 7 years	[107]
Male and Female Children	12 and 32 weeks gestation	2.4 μg BPA/g creatinine	Median urine concentration	Increased wheeze, respiratory tract infection and bronchitis risk from 6 months–7 years old; no change in atopy/IgE levels	[108]
Male and Female Children	16 weeks gestation, 26 weeks gestation and birth	2.4 μg BPA/g creatinine	Median urine concentration	Decreased lung function at 4 years, but not 5 years; 16 week BPA only: increased wheeze and persistent wheeze risk	[109]

BPA: bisphenol A; TSLP: thymic stromal lymphopoietin.
